# Loss resilience for two-qubit state transmission using distributed phase sensitive amplification

**DOI:** 10.1038/srep16296

**Published:** 2015-11-12

**Authors:** James M. Dailey, Anjali Agarwal, Paul Toliver, Nicholas A. Peters

**Affiliations:** 1 Applied Communication Sciences, 331 Newman Springs Road, Red Bank, New Jersey 07701, USA

## Abstract

We transmit phase-encoded non-orthogonal quantum states through a 5-km long fibre-based distributed optical phase-sensitive amplifier (OPSA) using telecom-wavelength photonic qubit pairs. The gain is set to equal the transmission loss to probabilistically preserve input states during transmission. While neither state is optimally aligned to the OPSA, each input state is equally amplified with no measurable degradation in state quality. These results promise a new approach to reduce the effects of loss by encoding quantum information in a two-qubit Hilbert space which is designed to benefit from transmission through an OPSA.

Optical fibres are the most compelling technology for transmitting quantum states, though loss severely constrains throughput and reach. Optical phase-sensitive amplifiers (OPSAs) can increase a signal quadrature’s amplitude, while adding the minimum allowable noise^1^,^2^ and have been used to improve transmission performance in classical communications^3^. Theoretical results show that distributing the OPSA along the transmission link logarithmically improves noise performance relative to lumped amplification^4^ and improves channel capacity for coherent state inputs^5^. Here, we consider the possibility of harnessing these benefits for improved quantum state transmission. We present the first experiment where we send pairs of non-orthogonal, phase-encoded 2-qubit quantum states through a distributed OPSA to preserve the transmission probability of encoded quantum states, thus beating direct transmission. We show experimentally that the input state quality, measured by two-photon interference visibility, can be maintained after transmission through the OPSA operating in the low-gain regime to offset intrinsic fibre loss.

In contrast to high-gain amplification resulting in multi-photon generation^6^, the results reported here build on our previous work demonstrating the first distributed OPSA utilizing constructive interference^7^. This interference arises from the coherent sum of probability amplitudes of photon-pair generation in the transmitter and the distributed OPSA, and relies on the indistinguishability of the two sources of photon-pairs^8^. In our previous work^7^, we demonstrated the transmission of quantum states optimally phase-aligned to the maximum gain of a distributed OPSA. Here we detail an advance beyond our earlier work where we now study the impact of a novel phase-encoding on the qubit’s constituent time-bins. Specifically, we demonstrate the transmission of *multiple two-qubit states* over a fibre-based distributed OPSA. Our results here are significant as they demonstrate that the OPSA supports successful transmission of a larger two-qubit quantum state space than what was demonstrated earlier^7^, even without changing the OPSA configuration. These advances promise a new approach to mitigate the effects of loss by encoding quantum information in a two-qubit Hilbert space designed to benefit from transmission through an OPSA, with potential impacts in the fields of quantum computing and quantum communications. In this paper, we present new data and build on the preliminary results previously reported^9^.

## Results

**Figure 1:**
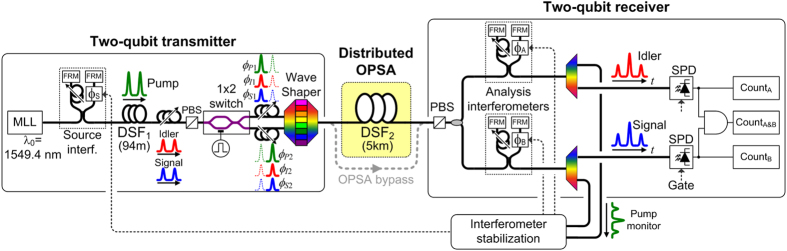
Experimental setup. MLL: Mode-locked laser, FRM: Faraday rotator mirror, DSF: dispersion-shifted fibre, PBS: polarization beam splitter, OPSA: Optical phase-sensitive amplifier, SPD: Single photon detector.

The experimental setup is shown in Fig. 1. A two-qubit transmitter generates phase-encoded states that are transmitted through a 5-km long distributed OPSA followed by a two-qubit receiver for state analysis. There are two distinct two-photon interference effects in this system, and it is important to consider how they arise and are used. The first interference effect was noted above, and is between the two-photon state amplitudes injected by the transmitter into the OPSA and the amplitude for pair generation in the OPSA. This effect is controlled at the transmitter by configuring the relative phases between the input two-photon state (signal and idler photons) and the OPSA pump, as well as by setting the pump intensity appropriately. The second interference effect arises from entanglement between the signal and idler photons. The entanglement-based interference is observed at the receiver by a two-photon time-bin interference fringe measurement. This measurement is made to characterize the two-qubit quantum states before and after transmission through the OPSA-based channel.

At the transmitter, changing the relative phase between the pump and the injected two-photon states can result in either amplification or de-amplification of the photon pairs sent into the OPSA channel^7^. This interference effect has also been considered previously, but where short, negligible-loss, nonlinear crystals^6^,^10^,^11^,^12^,^13^,^14^ or rubidium vapour cells^15^,^16^ were employed. Here we use a 5-km long dispersion-shifted fibre (DSF) with non-negligible loss and weak instantaneous gain to compensate for the distributed fibre loss, in order to limit the probability of higher-order photon number creation. First, the OPSA configuration is fixed by setting the pump characteristics (intensity, wavelength) which serves as a phase reference for the experiment. Next, the transmitter is used to set the signal and idler phases of each time-bin, relative to the pump, to produce a two-qubit state of the form:


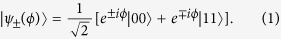


For 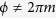, where 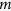 is an integer, these two states are symmetrically distributed about the pump-phase-aligned optimally-amplified state 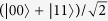 and are equally affected by the OPSA, albeit with a lower gain than for the optimal state. Note that in our earlier work^7^, the input quantum state was set to this pump-phase-aligned state. In contrast, in this experiment, we modulate the input quantum state by changing a phase 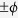 of the two-qubit state, leading to two-qubit states that are symmetrically offset from the pump-phase-aligned state. As 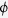 increases from zero, each two-qubit state 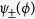 experiences decreasing levels of gain for a fixed pump phase. For 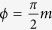 (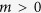), there will be no amplification, and at slightly larger phases, de-amplification occurs. This is consistent with the phase-sensitive nature of the OPSA, which was shown previously by measuring the gain of the coincidence counts as a function of changing the OPSA pump phase relative to the input photon pair phase^7^. These results indicate that even without modifying the OPSA pump properties, the OPSA channel can transmit all states with 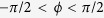 to reduce the impact of loss through constructive interference. To demonstrate the efficacy of such an encoding, we set 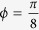, so the transmitted states are *maximally non-orthogonal* in that their fidelity is 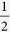, yielding:


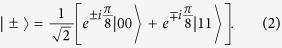


Before discussing the two photon interference (TPI) measurements, we present results on the photon pair flux as measured by the coincidence counting rates after transmission through the OPSA, when it is both active and not active (direct transmission). Note that for these measurements, the analysis interferometers are temporarily removed from the setup. For this measurement, the pump power and phase settings used in the OPSA are described in detail in the Methods section. First the direct transmission case is measured by blocking the pump at the OPSA input, so it acts only as a passive transmission fibre, yielding 159 ± 13 coincidences in 50 s. Next the pump is unblocked and the coincidence rate is 266 ± 16 per 50 s. The measured signal-idler coincidence rates are 2.2 ± 0.4 dB greater when the OPSA is active. Inclusion of an OPSA leads to coincidence counting rates that exceed what is possible via direct transmission by more than 5 standard deviations, and the intrinsic fibre loss is approximately compensated for input states 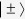.

Next we discuss the relative magnitude of the spontaneous emission in the OPSA. The probability of spontaneous generation in the 5-km distributed OPSA channel is measured by setting the signal and idler probability amplitudes from the source to zero in both time-bins using the Waveshaper. For the same OPSA pump power established above, which approximately compensates for the fibre loss, the measured coincidence counting rate due to spontaneous emission in the OPSA is smaller by a factor of 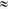
14 compared to the coincidence counting rate when the two-qubit transmitter signals are enabled.

**Figure 2:**
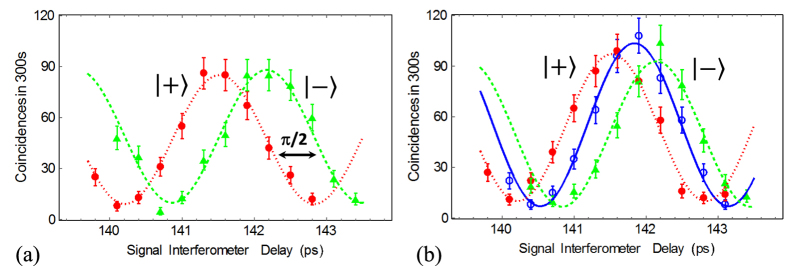
Two-photon interference fringe measurements for the two non-orthogonal states |+〉 and |−〉. (**a**) shows the measurement results after the transmitter, with raw visibilities of 81% ± 5% for both states. (**b**) shows the measurement results after transmission through the 5-km distributed OPSA with raw visibilities of 86% ± 4% and 81% ± 4%. Also shown in 2(**b**) is the fringe for a state that is optimally aligned with the OPSA pump phase: 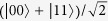 with raw visibility 87% ± 4%.

Having characterized the OPSA, we now describe the two-qubit state characterization measurements performed before and after transmission through the distributed OPSA. For state characterization, the analysis interferometers are inserted at the receiver. The analysis is carried out with a two-photon time-bin state analysis system comprised of a polarizing beam splitter to suppress cross-polarized Raman noise, a passive beam splitter, two imbalanced analysis interferometers matched to the pump interferometer, optical filters, single photon detectors, and coincidence counting circuitry. We note that while the choice to use a passive splitter sends half of the signal photons to the idler analysis arm (and vice versa), which are not measured, one could add additional detectors to measure them. To sweep out the TPI curve for 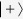 and 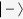, a small delay that applies a phase 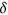 between the long and short paths of one analysis interferometer is varied. This delay is short relative to the time bin spacing, the detector gate width and the pulse width. This measurement has the effect of making the following projection: 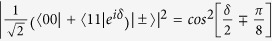, from which one can see the two non-orthogonal states have minima spaced by 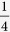 of a fringe period. The TPI measurements of the two maximally non-orthogonal input states before the OPSA are shown in Fig. 2(a) and have the expected behaviour. Each raw data point is the result of counting for 300 s and accidental coincidences have not been subtracted. The coincidence counting data is fit using the following function to extract the visibility (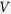): 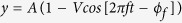, where 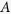 is the amplitude, 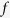 is the frequency, 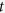 is the interferometer delay, and 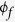 is the phase. The extracted visibilities of the 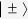 input states are the same within error and are 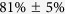. Note that the plots shown with solid circles and solid triangles in Fig. 2(a) correspond to the TPI measurements of the *two different non-orthogonal quantum states* and not to two measurement bases. Here our goal is to demonstrate that the transmitted states retain the qualities of the input states, namely that the maxima, minima, and phase encodings are substantially preserved as opposed to witnessing entanglement as in^7^.

Next, the two states are analysed after transmission through the OPSA. The states 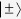 are created and sent through the 5-km long OPSA, after which they are characterized by a two-photon interference measurement shown in Fig. 2(b). The extracted raw visibilities are 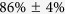 and 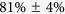. To within error of the experiment, both the visibility and the counting rates are the same before and after transmission though the distributed OPSA. Note that if the OPSA pump is turned off, the maximum coincidence rate drops due to the 5-km long DSF fibre loss. Next we measure the visibility for a state that is optimally phase-aligned to the OPSA pump phase, which corresponds to the state 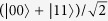. This is also shown in Fig. 2(b). This state, which was the state transmitted through a distributed OPSA in our previous work^7^, is offset by 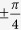 radians from the two non-orthogonal states, as expected. The phase-aligned state has a slightly higher maximum coincidence counting rate due to its alignment with the OPSA gain peak and the visibility obtained through curve-fitting is 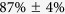. Our results demonstrate that the distributed OPSA supports the transmission of both non-orthogonal states equally with no measurable degradation in the state visibility while improving the transmission probability compared to direct transmission. To our knowledge, this is the first time encoded quantum states have been transmitted with greater probability than is possible by direct transmission.

While the present work focuses on a 5-km OPSA-based transmission channel, its scalability is also being analysed. Initial theoretical assessments of the input-output quantum state fidelity for coherent states suggest distributed OPSAs continue to provide benefits at longer distances^17^. Though we observe no measurable degradation in our experiment, the phase-sensitive amplifier will add noise from vacuum fluctuations. This noise will linearly increase with the fibre loss and may ultimately constrain the maximum transmission distance.

In conclusion, we demonstrate that a distributed OPSA can improve the transmission probability of non-orthogonal two-qubit quantum states with no measurable degradation in state quality as quantified by the two-photon interference visibility. This work presents a novel way to encode and transmit a phase in a single-photon pair to mitigate the impact of loss. The OPSA-enabled transmission probability exceeds what is possible via direct transmission by more than 5 standard deviations. While this work focuses on utilizing optical fibre, our approach could also find use in other platforms. For example, OPSAs can be realized on a photonic integrated circuit platform, which may be a critical enabling technology for quantum computing. Our encoding and amplification scheme could enable high-efficiency quantum state routing in such systems.

## Methods

The experiment shown in Fig. 1 is comprised of a two-qubit transmitter, a distributed OPSA, and a two-qubit receiver, each of which we describe below. The transmitter consists of a dispersion-shifted fibre (DSF)-based time-bin entangled photon pair source^18^,^19^,^20^. Two time-delayed copies of pump pulses from a mode-locked laser centred at 1549.4 nm with a 47-MHz repetition rate are created using a fibre-Michelson interferometer with 5 ns path-length mismatch. These pulses pump a 94-m long DSF to produce time-bin entangled signal-idler photon pairs through spontaneous four wave mixing. The 94-m long DSF is cooled in a bath of liquid nitrogen to reduce Raman scattering^21^. The signal and idler pairs are selected ±400 GHz (±3.2 nm) from the pump wavelength, and thus all are in the lowest-loss telecom transmission window. The pump centre wavelength is chosen to be close to the zero-dispersion wavelength of the DSF in the source and the OPSA to maximize the four-wave mixing efficiency.

After pair creation in the DSF, the output is encoded to create one of the two-qubit non-orthogonal states. A fibre polarization controller and polariser are used to suppress cross-polarized Raman noise from the source and also align the polarization to a 1 × 2 optical switch. The 1 × 2 optical switch is a high-speed electro-optic modulator. An electronic gating signal synchronized to the optical input triggers the switch to temporally demultiplex the two time-bins onto a pair of spatially distinct output fibres. The two fibres are sent into two different input ports of a Finisar Waveshaper 4000 S that can independently adjust the amplitude and the relative phases between the signal (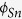), idler (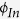), and pump (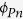) wavelengths in each of the time bins (labelled with 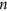). The Waveshaper recombines the two time-bins onto a single fibre once appropriate phase-shifts have been applied to create the desired state (see Eq. (2)). The output of the Waveshaper is connected to the 5-km long room-temperature OPSA and the analysis receiver. The classically measured loss of the 5-km DSF is 1.5 dB, of which approximately 0.2 dB is attributed to connector loss. The pump is spectrally demultiplexed after the analysis interferometers and used to lock the phase reference frame of both analysis interferometers to the source interferometer as described elsewhere^22^.

Now we describe how the OPSA pump phase and intensity are adjusted in the Waveshaper to set the OPSA gain. This is done with the analysis interferometers removed to avoid their lumped loss and eliminate the two-photon interference that would otherwise result from the time-bin entanglement. The pump, signal, and idler phases are first adjusted in the Waveshaper to obtain maximum gain in the OPSA (initially both time bins have the same settings) for the state 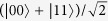. Next, the pump power is adjusted so that the OPSA two-photon gain compensates for the two-photon loss (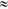
2.6 dB) due to the intrinsic fibre propagation loss. The total average pump power injected into the OPSA is −38.5 dBm, which is 35 dB below the average pump power used to spontaneously create time-bin entangled photon pairs in the transmitter. At this point, the pump power and pump phase are established and remain fixed for the remainder of the experiment. The desired non-orthogonal 2-qubit state (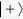 or 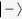) is then set in the transmitter using the Waveshaper to adjust the signal and idler relative phases in each time-bin to create the 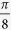 phase shifts indicated above in Eq. (2).

## Additional Information

**How to cite this article**: Dailey, J. M. *et al*. Loss resilience for two-qubit state transmission using distributed phase sensitive amplification. *Sci. Rep*. **5**, 16296; doi: 10.1038/srep16296 (2015).
